# Infantile fibrosarcoma with TPM3-NTRK1 fusion in a boy with Bloom syndrome

**DOI:** 10.1007/s10689-020-00221-1

**Published:** 2020-11-21

**Authors:** Sue M. Huson, Timo Staab, Marta Pereira, Heather Ward, Roberto Paredes, D. Gareth Evans, Daniel Baumhoer, James O’Sullivan, Ed Cheesman, Detlev Schindler, Stefan Meyer

**Affiliations:** 1 Department of Genetic Medicine, St Mary’s Hospital, Central Manchester Foundation Trust, Manchester, UK; 2 Department of Human Genetics, University of Würzburg, Würzburg, Germany; 3 Stem Cell and Leukaemia Proteomics Laboratory, School of Cancer and Imaging Sciences, The University of Manchester, Manchester Academic Health Science Centre, Manchester, UK; 4 Institute for Medical Genetics and Pathology, University Hospital Basel, Basel, Switzerland; 5 Department of Paediatric Histopathology, Royal Manchester Children’s Hospital, Central Manchester Foundation Trust, Manchester, UK; 6 Departments of Paediatric Haematology Oncology, Royal Manchester Children’s Hospital, Central Manchester Foundation Trust, Manchester, UK; 7 Academic Unit of Paediatric and Adolescent Oncology, University of Manchester, c/o Young Oncology Unit, The Christie NHS Foundation Trust, Wilmslow Road, Manchester, M20 6XB UK

**Keywords:** Bloom syndrome, Infantile fibrosarcoma, Cancer predisposition, TPM3-NTKR1 fusion

## Abstract

Bloom syndrome (BS) is a genomic and chromosomal instability disorder with prodigious cancer predisposition caused by pathogenic variants in *BLM*. We report the clinical and genetic details of a boy who first presented with infantile fibrosarcoma (IFS) at the age of 6 months and subsequently was diagnosed with BS at the age of 9 years. Molecular analysis identified the pathogenic germline *BLM* sequence variants (c.1642C>T and c.2207_2212delinsTAGATTC). This is the first report of IFS related to BS, for which we show that both *BLM* alleles are maintained in the tumor and demonstrate a TPM3-NTKR1 fusion transcript in the IFS. Our communication emphasizes the importance of long-term follow up after treatment for pediatric neoplastic conditions, as clues to important genetic entities might manifest later, and the identification of a heritable tumor predisposition often leads to changes in patient surveillance and management.

**Electronic supplementary material:**

The online version of this article (10.1007/s10689-020-00221-1) contains supplementary material, which is available to authorized users.

## Introduction

Bloom syndrome (BS, OMIM #210900) is an autosomal recessively inherited genomic and chromosomal instability disorder with pre- and postnatal growth failure and skin photosensitivity, characteristically manifesting with a butterfly-like shaped facial rash [1]. Other skin changes include poikiloderma and pigmentation anomalies, which often appear later in life. Additional BS features comprise immunodeficiency, endocrine problems, chronic obstructive lung disease and exceptional predisposition to malignancies. BS is caused by pathogenic variants in the *BLM* gene, encoding a RecQ helicase [1]**.** The *BLM* mutation spectrum in BS patients is well defined, and founder mutations have been identified in distinct ethnic groups. Cancer in BS comprises epithelial, hematological, mesenchymal and embryonal tumors, and is documented in more than 90% of the patients reported to the BS registry with a cancer prevalence of 33.4% by age 25 and 80.9% by age 40 [2]. Infantile fibrosarcoma (IFS) is an uncommon spindle cell tumor originating from soft tissue, accounting for approximately 10% of all childhood sarcomas, and mostly arising at the extremities [3]. While the etiology of IFS is poorly understood, the recurrent acquired chromosomal translocation t(12;15)(p13;q26), which causes oncogenic fusions involving the **ET**S ***v***ariant gene **6** (*ETV6*) and the neurotrophic tyrosine receptor kinase type 3 gene (*NTRK3*) has been identified in at least 70% of cases [4]. *NTRK* gene fusions give rise to constitutively active transcription and continuous production of TRK fusion proteins that stimulate cell growth and survival [4]. Here we report on a boy with IFS who was subsequently found to have BS.

## Clinical summary, methods and results

**Fig. 1:**
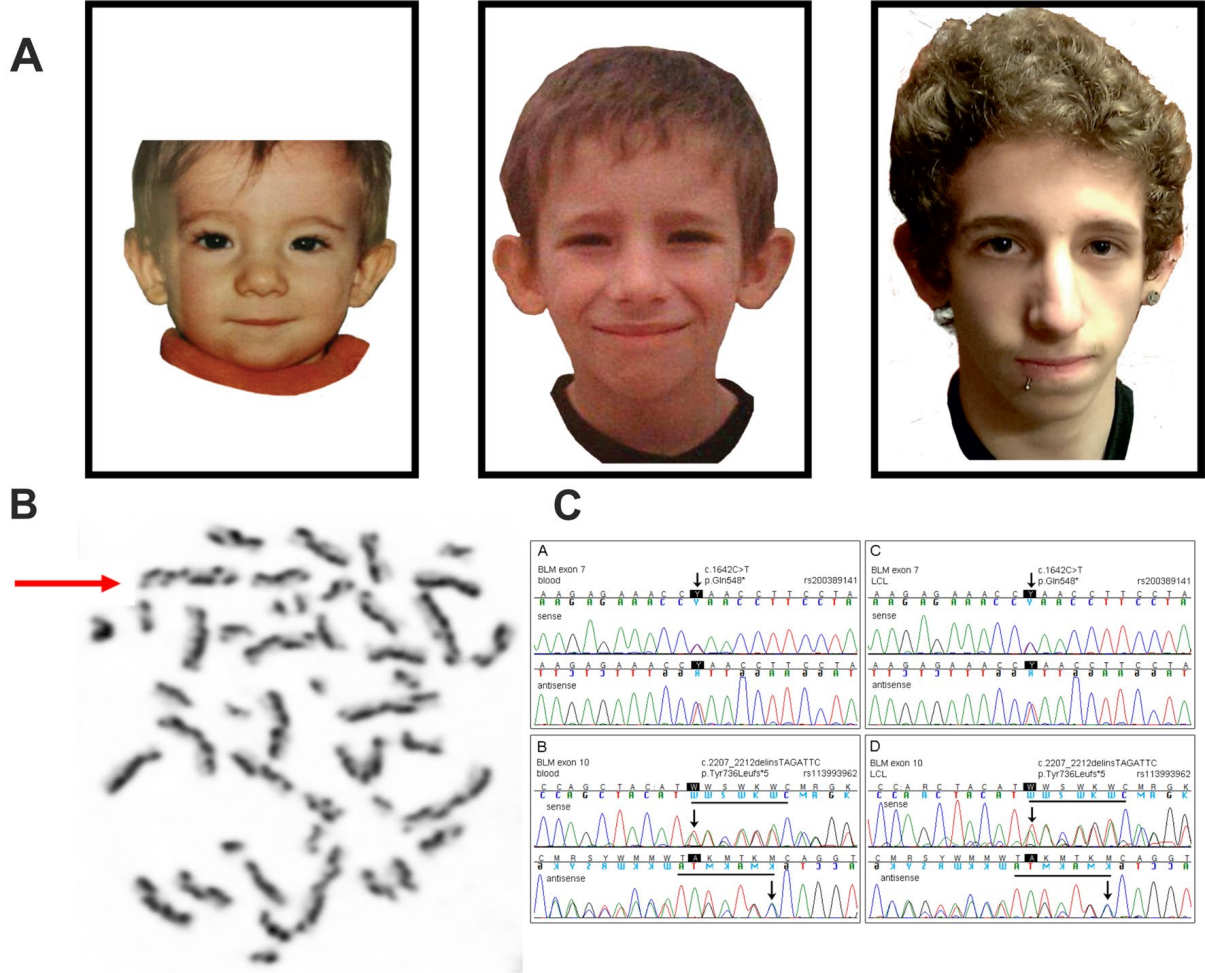
Bloom syndrome in a male patient**. a** Described patient with development of facial characteristics of Bloom syndrome. Unremarkable appearances aged three (left), and increasingly recognisable oval shaped face with nasal prominence and small chin age nine (middle) and 15 (left). **b** Karyotype with characteristic harlequin chromosomes (red arrow for example) in a metaphase from a PHA-stimulated peripheral blood lymphocyte culture exposed to BrdU, indicating a greatly increased rate of sister chromatid exchanges (SCEs). **c** Pathogenic *BLM* variants in peripheral blood lymphocyte and lymphoblastoid cell line DNA from the BS patient

**Fig. 2:**
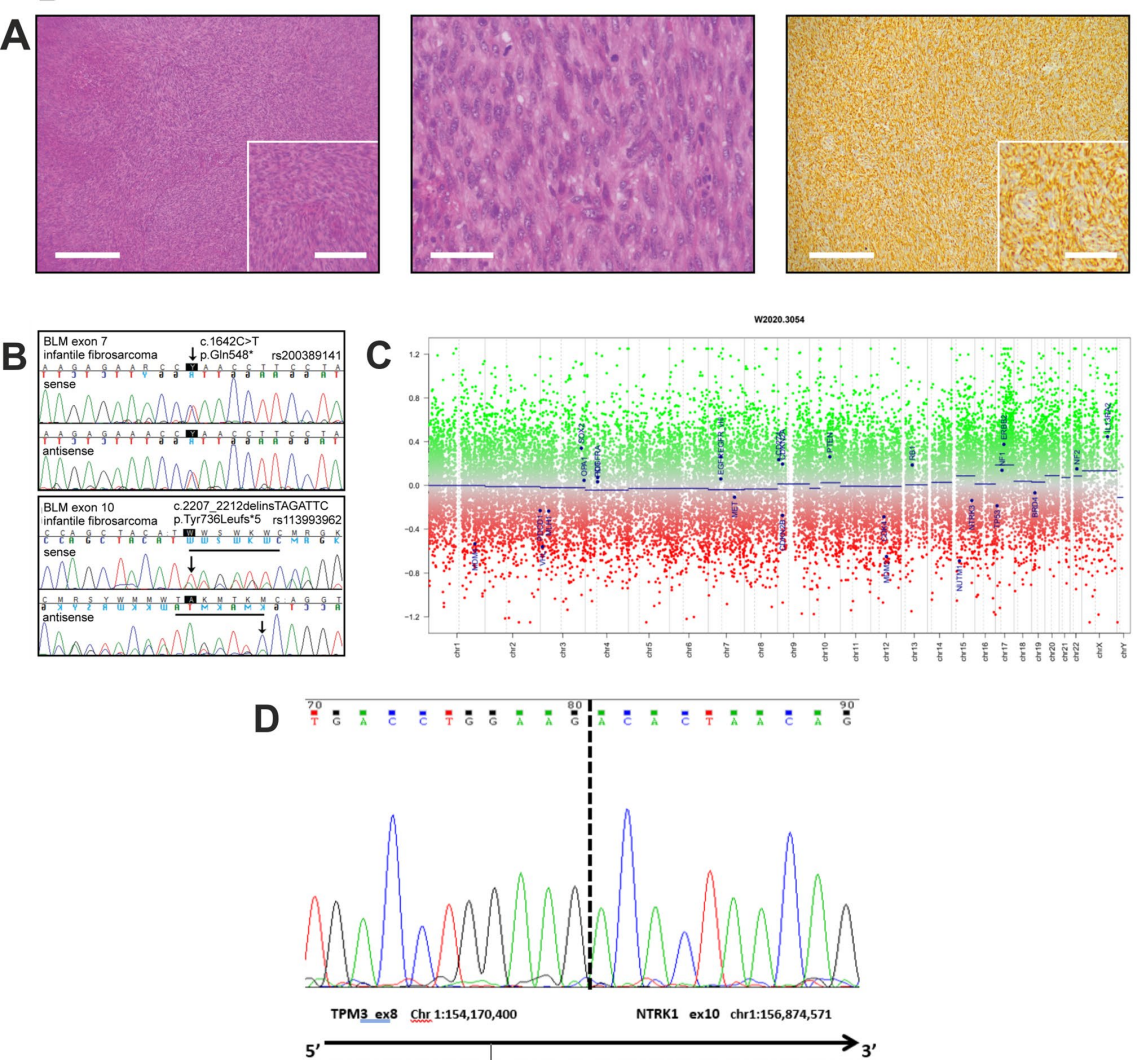
Infantile fibrosarcoma with TMP3-NTRK1 fusion in the boy with Bloom syndrome***. a*** Classical histological appearances of infantile fibrosarcoma with fascicles of uniform spindle cells (left panel), moderate nuclear pleomorphism and brisk mitotic activity (middle), positive for vimentin (right), but negative for markers of skeletal muscle, smooth muscle and neurogenic differentiation (left and right panel × 4, scale bar 500 µm; insert × 8, scale bar 200 µm; middle panel × 10, scale bar 100 µm). **b** Detection of both pathogenic variants as in Fig. 1c in fibrosarcoma DNA. **c** Copy number variation profile of the IFS. Depiction of chromosome 1 to 22, X and Y. Gains/amplifications are represented by positive (red), losses by negative (green) deviations from the baseline. Medians of segments are shown as blue lines. Flat profile with no evidence of gross copy number aberrations, arguing against genomic scars usually observed with Homologous Recombination Deficiency (HRD, “BRCAness”). The high scatter range of the individual signals indicates marked DNA degradation due to aged FFPE tumor material. 26 brain tumor relevant gene regions are highlighted for easier assessment (see Hovestadt & Zapatka, http://www.bioconductor.org/packages/devel/bioc/html/conumee.html). **d** Sanger sequencing analysis of the *TPM3-NTRK3* gene fusion illustrating that the fusion occurred between TPM3 NM_152263 exon 8 and NTRK1 NM_002529 exon 10 (with chromosomal position for both fused genes) in a tail-to-tail manner (arrow indicating 3′ to 5′ direction)

A small (2nd centile) and microcephalic (0.4th centile) boy (Fig. 1a) was investigated at the age of 6 months for a localised soft tissue swelling at the right forearm in late 2000. He is the only child of a white British couple with no significant family cancer history, albeit a maternal half-sister later developed an astrocytoma in her teens. The boy had several areas of uneven, subtly increased skin pigmentation (“café-au-lait” spots with irregular outline). The tumor of his forearm was completely resected. Histological features were consistent with IFS (Fig. 2a). On follow up over the next several years the boy continued to grow poorly (< 2nd centile, with normal height parents), and developed more non-uniform pigmented skin changes. Initially only subtle facial features with long and narrow face, small lower jaw and prominent nose became more apparent over time (Fig. 1a). In view of the IFS in infancy, poor growth, and microcephaly with facial conspicuousness further studies were initiated at the age of 9 years. DNA repair disorders, including BS, were among the top candidates for genetic investigations. Sister chromatid exchange (SCE) and karyotype analysis was carried out on the boy’s peripheral lymphocytes using standard protocols (see supplemental material for further details) [5]. Germline mutational analysis of *BLM* was performed with genomic DNA from peripheral blood using primers and Sanger technology as previously published [5]. Tumor tissue from FFPE specimens of the IFS was reviewed and examined by FISH for the presence of the ETV6-NTRK3 and the LMNA-NTRK1 fusions. Tumor DNA isolated from macro-dissected material, enriched for neoplastic cell content, was subjected to somatic *BLM* sequencing and copy number variation (CNV) analysis. RNA from macro-dissected FFPE tumor tissue was targeted-enriched on an RNA custom panel designed for sarcoma and CNS tumors [6]. After library preparation, NGS and de-multiplexing, FASTQ files were analysed using a customised version of the Detect QIAseq RNAscan Fusions workflow in the CLC Genomics Workbench 12 analysis software (see Supplemental Material for more details).

Studies of peripheral lymphocytes revealed a markedly increased rate of spontaneous chromosome breakage with the distinct morphology of harlequin-like chromosomes (Fig. 1b), caused by greatly increased rates of SCEs (maximum > 50/cell vs. 5 − 6/cell in normal controls), and quadriradial configurations of homologous chromosomes (in 1.4% of metaphases), both indicative of the cellular BS phenotype. Sequencing of the *BLM* gene in blood identified two heterozygous pathogenic variants (Fig. 1c). The nonsense (stop) variant c.1642C > T with the predicted consequence p.(Gln548*) is listed with the dbSNP annotation rs200389141. As the second pathogenic variant the deletion/insertion c.2207_2212delinsTAGATTC with the deduced effect p.(Tyr736Leufs*5) was detected. This variant is listed with the dbSNP annotation rs113993962. Both mutations were confirmed in a patient-derived lymphoblastoid cell line. Unfortunately, parental DNA was not available to confirm that the two *BLM* variants were indeed located on different parental alleles proving compound heterozygosity. However, the notion of increased SCE rates in the patient’s PHA-stimulated blood T lymphocytes in culture and in the patient-derived B-lymphoblastoid cell line (29.1 ± 10.4 SCEs per metaphase, i.e. > 10 times increased compared to a normal control studied in parallel, which revealed 2.2 ± 1.5 SCEs per metaphase) supports the concept that the *BLM* variants are bi-allelic and that way disease-causing. Sequence analysis of DNA isolated from tumor tissue also identified both pathogenic *BLM* variants (Fig. 2b), implying that both *BLM* alleles were retained in this IFS. Enhanced copy-number variation analysis was performed using an Illumina DNA methylation array and the conumee package with tumor DNA (https://bioconductor.org/packages/release/bioc/vignettes/conumee/inst/doc/conumee.html). Copy number profiling showed signs of marked DNA degradation in the IFS due to the age of the paraffin block, but overall revealed a flat profile with no evidence of gross copy number aberrations, arguing against genomic scars usually observed in Homologous Recombination Deficiency (HRD, “BRCAness”) (Fig. 2c) Fluorescent in situ hybridisation (FISH) analysis of the tumor was negative for ETV6-NTRK3 and LMNA-NTRK1 fusions (data not shown). However, an RNA-based assay using a newly designed sarcoma panel including NTRK fusion probes, carried out on tumor tissue 19 years after the IFS resection, uncovered a fusion between the Tropomyosin 3 gene (*TPM3*) and the Neurotrophic Receptor Tyrosine Kinase 1 gene (*NTRK1*). The TPM3-NTRK1 fusion occurred between TPM3 NM_152263 exon 8 and NTRK1 NM_002529 exon 10 in a tail-to-tail manner (Fig. 2d). On clinical follow up the 20-year-old man is of short stature and has multiple skin pigment abnormalities. He is under regular surveillance for complications of BS. There has been no recurrence of the IFS and no occurrence of any other tumors.

## Discussion

In the individual reported here the presentation of IFS, poor growth during childhood, microcephaly and progressive skin abnormalities, but not the characteristic facial rash, led to the diagnosis of BS. The absence of the typical facial rash—as holds for the presented case—was noted in several BS patients, among them an individual with BS presenting later in life with nasopharyngeal carcinoma [7]. Multiple neoplasms have been described with BS, but no preferential causation of distinct malignancies has been observed [2]. The malignancies in BS follow a similar distribution as in the general population but at a much younger age. Only about 4% of the recorded malignancies are of mesenchymal (sarcomas) or embryonal (blastomas or germ cell tumors) origin [2], and there is no obvious correlation between mutation type and malignancy. The *BLM* variant c.1642C > T has first been reported as syndrome-causing and multiplicatively recurring mutation by German et al. in two patients of European and North American descent (foundred 12) [8]. It has been re-described in a British patient with ALL and therapy-related AML [5]. More recently, it was classified among deleterious germline *BLM* mutations with moderately increased risk for early-onset colorectal cancer [9]. There are five entries of the c.1642C > T variant in the Global Variome shared LOVD, including a locus-specific database for *BLM* [https://databases.lovd.nl/shared/genes/BLM], designated as BLM_000052. They represent patients from the Netherlands, while others locate the origin of this mutation with the effect Gln548* to Eastern Europe, being more frequent in the Slavic populations with a carrier frequency of 0.1% [2]. The c.2207_2212delinsTAGATTC variant is the predominant pathogenic *BLM* variant identified in Ashkenazi Jews, a 6-bp deletion/7-bp insertion in exon 10 of *BLM*, often (for brevity) designated blm^Ash^, which has recently been described in a BS patient with nasopharyngeal carcinoma later in life [7]. Germline genetic variation conferring predisposition to IFS, or contributing to IFS etiology, is not known [3]. Given the extreme unlikeliness of both conditions occurring together, a causative relationship of IFS with BS must be considered. The way in which BS paves the way to cancer is understandably defective DNA damage response resulting in genomic instability in a way not involving HRD (BRCAness) as a mechanism as documented in the present case. Closer insights in which way BLM-related genomic instability could facilitate tumor progression in BS patients, and in therapeutic options may be identified through the identification of the mutational signature(s) of BS-related tumors, which is yet unreported and unfortunately was inconclusive here due to technical problems with DNA degradation. In this case BLM-related genomic instability gave rise to an IFS negative for the common ETV6-NTRK3 and the rarer LMNA-NTRK1 fusions but harboring* a* TPM3-NTRK1 fusion, highlighting that RNA sequencing is capable of identifying all NTRK fusions without prior knowledge of the breakpoint or partner [6]. The tropomyosin receptor kinase [TRK] fusion protein stimulates cell proliferation and survival pathway activation. TPM3-NTRK1 fusions have been described before in IFS [10], and other malignancies [11]. NTRK fusion-proteins in IFS and numerous adult and paediatric tumour types have emerged from a promising therapeutic target to the most targetable of oncogenic drivers [12]. Long term follow up of IFS, as for other cancers in childhood, is important, as syndromic associations, which might allow for specific surveillance and management strategies and might have also implications for the wider family with respect to cancer risk [9], may be subtle and only manifest later in life.

## Electronic supplementary material

Below is the link to the electronic supplementary material.

Supplementary file1 (DOCX 22 KB)

## Abbreviations

BS: Bloom syndrome
BLM: Bloom syndrome helicase gene or protein
IFS: Infantile fibrosarcoma
SCE: Sister chromatid exchange
