# Supplementary material for: Infantile fibrosarcoma with TPM3-NTRK1 fusion in a boy with Bloom syndrome
Source: Fam Cancer. 2020 Nov 21;21(1):85–90. doi: 10.1007/s10689-020-00221-1 (PMC8799568; doi:10.1007/s10689-020-00221-1)
Supplement: Supplementary file 1 — Supplementary file1 (DOCX 22 KB) [file 10689_2020_221_MOESM1_ESM.docx]

**SUPPLEMENTAL MATERIAL**

***Huson et al.* Infantile fibrosarcoma with TPM3−NTRK1 fusion in a boy with Bloom Syndrome**

**Sister chromatid exchange and karyoptype analysis**

Sister chromatid exchange (SCE) and karyotype analysis was carried out using standard protocols. In brief, four phytohemagglutinin (PHA)-stimulated whole blood cultures were established from the proband and age-matched controls, respectively. Two unsychronized cultures were exposed to bromodeoxyuridine (5-bromo-2'-deoxyuridine, BrdU) at a concentration of 10 mg/ml, one unsychronized culture had no BrdU and the fourth culture was synchronized with thymidine and deoxycytidine (2'*-*deoxyuridine, dU). All of the cultures were harvested after 72 h. Metaphases obtained from the BrdU-treated cultures were exposed to UV and 2xSSC for 12 min each and then stained for 2 min in Giemsa. 50 cells from each of these cultures were analyzed to obtain the level of SCE. Metaphases obtained from the unsynchronized culture were solid stained with Giemsa. 50 cells were examined from this culture to obtain the level of spontaneous chromosome damage. Metaphases obtained from the synchronized culture were G-banded using Trypsin and Giemsa. This culture was analyzed to obtain the constitutional karyotype.

**Sanger sequencing**

The *BLM* gene was analysed for mutations using the following primers:

BLM_Ex1_F: gatgtccgagtgcgacagta (length: 20, Tm: 59,86 °C, GC%: 55)

BLM_Ex1_R: cagagcagggctagatcaatg (length: 21, Tm: 59,99°C, GC%: 52,38)

BLM_Ex2_F: agtccttcctcccctcaaaa (length: 20, Tm: 60,04°C, GC%: 50)

BLM_Ex2_R: tgcctatgtgacagcaaacc (length: 20, Tm: 59,72°C, GC%: 50)

BLM_Ex3_F: ggatccatctaatctagtttttcca (length: 25, Tm: 58,96°C, GC%: 36)

BLM_Ex3_R: tcccaatggctagctttgaa (length: 20, Tm: 60,71°C, GC%: 45)

BLM_Ex4_F: cttaatcgctcatgccctgt (length: 20, Tm: 60,24°C, GC%: 50)

BLM_Ex4_R: aaacaatttaaagtatcccagaaacaa (length: 27, Tm: 59,67°C, GC%: 25,93)

BLM_Ex5_F: ttgtctgatcagtggtagaaaaat (length: 24, Tm: 57,02 °C, GC%: 33,33)

BLM_Ex5_R: gccacaggttcaaaacacaa (length: 20, Tm: 59,59°C, GC%: 45)

BLM_Ex6_F: caccatgcctagccaagact (length: 20, Tm: 60,28°C, GC%: 55)

BLM_Ex6_R: ttccagaagggggaaaatta (length: 20, Tm: 58,48°C, GC%: 40)

BLM_Ex6_R (Neu): tgaacattttgccctgtccatt (length: 22, Tm: 51°C, GC%: 41)

BLM_Ex7_F: atttgcttttgtggcctacc (length: 20, Tm: 59,09°C, GC%: 45)

BLM_Ex7_R: ttttgggacagttttgttttca (length: 22, Tm: 59,51°C, GC%: 31,82)

BLM_Ex7_F (Neu): AACTACAGATTTGCTTTTGTGGC (length: 23, Tm: 52°C, GC%: 39)

BLM_Ex7_R (Neu): caatgttttcaggcaatgatgatt (length: 24, Tm: 51°C, GC%: 33)

BLM_Ex8_F: aaatgctaaagctgtactttcactg (length: 25, Tm: 58,37°C, GC%: 36)

BLM_Ex8_R: atggattcctgccaattcac (length: 20, Tm: 59,76°C, GC%: 45)

BLM_Ex9_F: tgctctgaagacagaacctga (length: 21, Tm: 58,74 °C, GC%: 47,62)

BLM_Ex9_R: aaaaaggttatccagaggactgaa (length: 24, Tm: 59,56°C, GC%: 37,50)

BLM_Ex10_F: caaatgtaattttgtcaggttaatgt (length: 26, Tm: 57,72°C, GC%: 26,92)

BLM_Ex10_F(Neu): CTGCCTGAGTTATGCTTAATAAGGGTT (Tm: 57°C)

BLM_Ex10_F(Neu1): ATGCTAGTCTTGAACTCCTGACCCC (Tm: 59°C)

BLM_Ex10_R: ttctggatgaaagttgtacaaaaa (length: 24, Tm: 57,92°C, GC%: 29,17)

BLM_Ex10_R(Neu): TGTACTGAGGTTATATGAGAGAATACGTTG (Tm: 58°C)

BLM_Ex11_F: tgatggaatttgaagaccacag (length: 22, Tm: 59,97°C, GC%: 40,91)

BLM_Ex11_R: tggtagtatgtatttatcggtatttcc (length: 27, Tm: 58,02°C, GC%: 33,33)

BLM_Ex12_F: cattgagcagtgttggcttt (length: 20, Tm: 58,93°C, GC%: 45)

BLM_Ex12_R: gccccagggttctcaaatac (length: 20, Tm: 60,69°C, GC%: 55)

BLM_Ex13_F: tgggggttaggattttaggg (length: 20, Tm: 60,01 °C, GC%: 50)

BLM_Ex13_R: tgctgtcataatgcaaaaagg (length: 21, Tm: 58,83°C, GC%: 38,10)

BLM_Ex14_F: cgggagatctatttatggttca (length: 22, Tm: 58,09°C, GC%: 40,91)

BLM_Ex14_R: cattctacatgtgcatgtttgg (length: 22, Tm: 58,97°C, GC%: 40,91)

BLM_Ex15_F: ccttcaagtctgtgccttatga (length: 22, Tm: 59,38°C, GC%: 45,45)

BLM_Ex15_F(Neu): GATGATAAGCAGTGGAGCAGGTAAGA (Tm: 58°C)

BLM_Ex15_R: tcatgaggctgaagatgacag (length: 21, Tm: 58,97°C, GC%: 47,62)

BLM_Ex15_R(Neu1): GAAAGGATACAAAGGAAACCAATAACTA (Tm: 54°C)

BLM_Ex15_R(Neu2): TTCTTAATCCAGATGGTAGTATATGACAGC (Tm: 58°C)

BLM_Ex16_F: tgatatgctctatttttccccta (length: 23, Tm: 57,06°C, GC%: 34,78)

BLM_Ex16_F(Neu): TATAAGTCAAACCATCATCATTGGGGATAAT (Tm: 56°C)

BLM_Ex16_R: ccaccttttgcaatctaatttgt (length: 23, Tm: 59,44°C, GC%: 34,78)

BLM_Ex16_R(Neu): AATTTAAAATATTCTACCTGCCCATGTTGGA (Tm: 56°C)

BLM_Ex17_F: ggcattgttaccttaattatagcaga (length: 26, Tm: 59,13 °C, GC%: 34,62)

BLM_Ex17_R: cacccactcagatgaactcg (length: 20, Tm: 59,26°C, GC%: 55)

BLM_Ex18_F: gcctcttctatttgagggtga (length: 21, Tm: 58,40°C, GC%: 47,62)

BLM_Ex18_R: ttttggttcactcattgtgagat (length: 23, Tm: 58,62°C, GC%: 34,78)

BLM_Ex19_F: aagcccctgtatgggtacaa (length: 20, Tm: 59,31°C, GC%: 50)

BLM_Ex19_R: ctgtgccacgtaacaaagga (length: 20, Tm: 59,76°C, GC%: 50)

BLM_Ex20_F: gtgctgaatgcgtgaatgag (length: 20, Tm: 60,42°C, GC%: 50)

BLM_Ex20_R: gcgtcgcttcacattaaaca (length: 20, Tm: 59,88°C, GC%: 45)

BLM_Ex21_F: ccagtgcgacatcacctgta (length: 20, Tm: 60,74 °C, GC%: 55)

BLM_Ex21_R: atccttcaaagcaaggcaga (length: 20, Tm: 59,96°C, GC%: 45)

BLM_Ex22_F: cgtaggcagaaaatgcacaa (length: 20, Tm: 59,87°C, GC%: 45)

BLM_Ex22_F(Neu): GGTGGGTTGGTCACAAGTCAGAAAATTA (Tm: 58°C)

BLM_Ex22_R: aaacaacggccacaacactt (length: 20, Tm: 60,45°C, GC%: 45)

BLM_Ex22_R(Neu): TGCCCCCTGGGATGTTTCTGACT (Tm: 59°C)

cDNA:

BLM_cDNA_3_F: TCGGAATAGGCAAGCTTCCGGC (Tm: 59°C)

BLM_cDNA_7_F: AATAGGCAAGCTTCCGGCGGGAA (Tm: 59°C)

BLM_cDNA_48_F: GGGAAGTTTGGATCCTGGTT (length: 20, Tm: 60,2°C, GC%: 50)

BLM_cDNA_120_F: ATCTACAGGAGCAACTAGAACG (length: 22, Tm: 53°C, GC%: 45)

BLM_cDNA_295_R: TGAAGGAAAAGTCTTCGGTAACA (length: 23, Tm: 60,2°C, GC%: 39)

BLM_cDNA_599_R: TGGGGTGGTGTAACAAATGA (length: 20, Tm: 59,7°C, GC%: 45)

BLM_cDNA_545_F: ACCATCAATGATTGGGATGA (length: 20, Tm: 58,6°C, GC%: 40)

BLM_cDNA_1280_R: AGCAGTTCGTTCCCACAATC (length: 20, Tm: 60,1°C, GC%: 50)

BLM_cDNA_990_F: CGGATTTTGTTCCACCTTCT (length: 20, Tm: 59°C, GC%: 45)

BLM_cDNA_1202_F: CAGCAGCAGCTTATTCATGTG (length: 21, Tm: 59,7°C, GC%: 48)

BLM_cDNA_1607_R: CTTGGTGTTTCAGCCCAGTT (length: 20, Tm: 60,15°C, GC%: 60,1)

BLM_cDNA_1849_R: GACCTTCCTTGATGGGTTGA (length: 20, Tm: 59,9°C, GC%: 50)

BLM_cDNA_1822_F: AGCCAGCAAATCTTCCACAG (length: 20, Tm: 60,4°C, GC%: 50)

BLM_cDNA_1984_F: GTCAGCACAAAATTTAGCATCCAG

BLM_cDNA_2127_R: TCTTCACCAAGCAGTGCAGCAT

BLM_cDNA_2313_R: GCTTCTGAGTCAGTCTTATCAC

BLM_cDNA_2392_R: TGCACAGATCTTTTCTGGAGTG (length: 22, Tm: 60,4°C, GC%: 45)

BLM_cDNA_2269_F: CCAAAAGCTGACTTCCTTGG (length: 22, Tm: 59,8°C, GC%: 50)

BLM_cDNA_2437_F: GGAGAATCTCTATGAGAGGAAG

BLM_cDNA_2478_R: CTGACACAATGTGCTTCATCAA

BLM_cDNA_2589_F: CCACAGCTAATCCCAGGGTA

BLM_cDNA_2732_R: GGTGCTTTCTGATCCATTCTAG

BLM_cDNA_2772_F: TAATTTACTGCCTCTCCAGGCG

BLM_cDNA_2820_R: AGCGAGCCCATCTCTCTGTA (length: 20, Tm: 60,1°C, GC%: 55)

BLM_cDNA_2707_F: GCCTAAAAAGGTGGCATTTG (length: 20, Tm: 59,6°C, GC%: 45)

BLM_cDNA_2939_F: GCATTTGGAATGGGGATTGA (length: 20, Tm: 50°C, GC%: 45)

BLM_cDNA_2993_R: AACCCTCCACAGATTTAGGGAG (length: 22, Tm: 55°C, GC%: 50)

BLM_cDNA_3133_F: CCATCATACAAGAGAAACTCAC (length: 22, Tm: 51°C, GC%: 41)

BLM_cDNA_3268_R: ATCACAAGAAACATCTGGGTGT (length: 22, Tm: 51°C, GC%: 41)

BLM_cDNA_3321_F: GAGATGTGACTGACGATGTGAA (length: 22, Tm: 53°C, GC%: 45)

BLM_cDNA_3401_R: ATCTTCCAGAAGGACCTACATG (length: 22, Tm: 53°C, GC%: 45)

BLM_cDNA_3435_R: CAAGAAAATGTCGACCAGCA (length: 20, Tm: 59,8°C, GC%: 45)

BLM_cDNA_3321_F: GAGATGTGACTGACGATGTGAAA (length: 23, Tm: 60,2°C, GC%: 43)

BLM_cDNA_3540_F: AGCTGATACTTGACAAGATTTTGG (length: 24, Tm: 52°C, GC%: 38)

BLM_cDNA_3581_R: ATCGCCTGGTCATTGGCATT (length: 20, Tm: 52°C, GC%: 50)

BLM_cDNA_3712_F: AGCAAAAGTGTCTCAGAGGGAA (length: 22, Tm: 53°C, GC%: 45)

BLM_cDNA_3763_R: CAGAGATTTGCAGACTTCTGTA (length: 22, Tm: 51°C, GC%: 41)

BLM_cDNA_3861_F: CTGATCCTGAGGTTTTGCTTC (length: 21, Tm: 52°C, GC%: 48)

BLM_cDNA_3907_R: CCGCACCATATTTTTCCAGT (length: 20, Tm: 59,8°C, GC%: 45)

BLM_cDNA_3781_F: TCTGGGGAAAGTTTTTGGTG (length: 20, Tm: 59,9°C, GC%: 45)

BLM_cDNA_3976_F: AGACAGTTCCCCAGGGATAA (length: 20, Tm: 52°C, GC%: 50)

BLM_cDNA_4129_R: AGCAGTTTTTCTCCTCTTAGAC (length: 22, Tm: 51°C, GC%: 41)

BLM_cDNA_4209_F: CGAAATCCTCCAGCATCATT (length: 20, Tm: 60°C, GC%: 45)

BLM_cDNA_4386_R: TGGTCAGATGCTGACAAACA (length: 20, Tm: 58,8°C, GC%: 45)

BLM_cDNA_4254+123_R: AAGAAGAACTATCACCCCCCAGCATTTATT (Tm: 59°C)

**Copy number profiling**

Enhanced copy-number variation analysis was performed using the Illumina Infinium Human Methylom EPIC Array with tumor DNA according to the manufacturer’s instructions. The array was used to extract information about copy-number alterations as initially described by Sturm et al. 2012 [1]. Copy number plots were generated through conumee (https://bioconductor.org/packages/release/bioc/vignettes/conumee/inst/doc/conumee.html) with the intensity values of the ‘methylated’ and ‘unmethylated’ channel combined [2].

**Gene fusion detection**

200 ng of RNA were extracted from FFPE tumor tissue using the RecoverAll™ Total Nucleic Acid Isolation Kit for FFPE (Thermo Fisher Scientific, Waltham, MA, USA). Targeted enrichment and library preparation was performed using a QIAseq Targeted RNAscan custom panel designed for sarcomas and CNS tumors (QIAGEN GmbH, Hilden, Germany) for Illumina, following the manufacturer’s protocols. Sequencing was carried out on a NextSeq 550 sequencer (Illumina Inc. San Diego, CA, USA), following the manufacturer’s protocols. Samples were de-multiplexed and FASTQ files generated using the BCL2FASTQ 2 programme. FASTQ files for each sample were then analysed using a customised version of the Detect QIAseq RNAscan Fusions workflow in the CLC Genomics Workbench 12 analysis software (QIAGEN GmbH, Hilden, Germany). The sequence across the breakpoint of the TPM3−NTRK1 fusion was confirmed by genomic Sanger sequencing.

**Sequencing primers for gene-fusion detection**

TPM3_ex8 CGTGCTGAGTTTGCTGAGAG

NTRK1 _ex10 AAGGAAGAGGCAGGCAAAGA

**References:**

1. Sturm D, Witt H, Hovestadt V, Khuong-Quang D-A, Jones DT, Konermann C, Pfaff E, Toenjes M, Sill M, Bender S, Kool M, Zapatka M, Becker N, Zucknick M, Hielscher T, Liu XY, Fontebasso AM, Ryzhova M, Albrecht S, Jacob K, Wolter M, Ebinger M, Schuhmann MU, Meter T van, Fruehwald MC, Hauch H, Pekrun A, Radlwimmer B, Niehues T, Komorowski G von, et al.: **Hotspot mutations in H3F3A and IDH1 define distinct epigenetic and biological subgroups of glioblastoma.** Cancer Cell. 2012, **22**:425–437.

2. Hovestadt V, Zapatka M. Conumee: Enhanced copy-number variation analysis using Illumina DNA methylation arrays. R package version 1.9.0, <http://bioconductor.org/packages/conumee/>.
